# Transient ureteral obstruction after mini-percutaneous nephrolithotomy is associated with stone volume and location: results from a single-center, real-life study

**DOI:** 10.1007/s00345-024-04832-6

**Published:** 2024-03-13

**Authors:** Marco Nizzardo, Stefano Paolo Zanetti, Andrea Marmiroli, Gianpaolo Lucignani, Matteo Turetti, Carlo Silvani, Franco Gadda, Fabrizio Longo, Elisa De Lorenzis, Giancarlo Albo, Andrea Salonia, Emanuele Montanari, Luca Boeri

**Affiliations:** 1 Department of Urology, Fondazione IRCCS Ca’ Granda – Ospedale Maggiore Policlinico, Milan, Italy; 2 Department of Clinical Sciences and Community Health, University of Milan, Via Della Commenda 15, 20122 Milan, Italy; 3 Division of Experimental Oncology/Unit of Urology, URI, IRCCS Ospedale San Raffaele, University Vita-Salute San Raffaele, Milan, Italy

**Keywords:** Percutaneous nephrolithotomy, Ureteral obstruction, Stone, Complications, Exit strategies

## Abstract

**Purpose:**

To evaluate the rate of and predictors of ureteral obstruction after mini-percutaneous nephrolithotomy (mPCNL) for kidney stones.

**Methods:**

We analyzed data from 263 consecutive patients who underwent mPCNL at a single tertiary referral academic between 01/2016 and 11/2022. Patient’s demographics, stone characteristics, and operative data were collected. A nephrostomy tube was placed as the only exit strategy in each procedure. On postoperative day 2, an antegrade pyelography was performed to assess ureteral canalization. The nephrostomy tube was removed if ureteral canalization was successful. Descriptive statistics and logistic regression models were used to identify factors associated with a lack of ureteral canalization.

**Results:**

Overall, median (IQR) age and stone volume were 56 (47–65) years and 1.7 (0.8–4.2) cm^3^, respectively. Of 263, 55 (20.9%) patients showed ureteral obstruction during pyelography. Patients without ureteral canalization had larger stone volume (*p* < 0.001), longer operative time (*p* < 0.01), and higher rate of stones in the renal pelvis (*p* < 0.01) than those with normal pyelography. Length of stay was longer (*p* < 0.01), and postoperative complications (*p* = 0.03) were more frequent in patients without ureteral canalization. Multivariable logistic regression analysis revealed that stone volume (OR 1.1, *p* = 0.02) and stone located in the renal pelvis (OR 2.2, *p* = 0.04) were independent predictors of transient ureteral obstruction, after accounting for operative time.

**Conclusion:**

One out of five patients showed transient ureteral obstruction after mPCNL. Patients with a higher stone burden and with stones in the renal pelvis are at higher risk of inadequate ureteral canalization. Internal drainage might be considered in these cases to avoid potential complications.

## Introduction

Urolithiasis is one of the main diseases that urologists have to manage during the everyday clinical practice. An epidemiological review shows a 1.7–14.8% prevalence of kidney stones from the general population in various countries, revealing an incremental trend during the last decades [1].

Different surgical modalities are available for the treatment of kidney stone with stone’s (size, location, density) and patient’s characteristics being used in clinical practice to select the best treatment option [2]. According to the European Association of Urology (EAU) guidelines, percutaneous nephrolithotomy (PCNL) is the gold standard treatment for kidney stone > 2 cm [3, 4].

PCNL is considered a safe and well-tolerated procedure; however, a specific set of complications are reported such as pain, urinary tract infections, urine leakage, urothorax and pneumothorax, bleeding, and post-procedural ureteropelvic junction (UPJ) obstruction [5, 6]. Among these, long-term upper urinary tract obstruction seems to be a rare event, localized at the proximal ureter and UPJ, documented in less than 1% of cases [5]. Conversely, the most frequently reported reason for transient post-PCNL urinary obstruction is local edema, due to the presence of impacted stones and mechanical trauma that occurred during the procedure and intracorporeal lithotripsy [5]. In addition to these factors, concurrent ipsilateral ureteroscopy, stone location, and type of surgical access could also contribute to a transient lack of ureteral canalization after PCNL [7]. To avoid obstructive complications, an indwelling ureteral stent is commonly placed as an exit strategy after standard procedures [8]. However, ureteral stents are associated with postoperative discomfort, and they require in-office removal [9].

Nowadays, the miniaturization of surgical instruments has progressively shifted PCNL into a tubeless or totally tubeless procedure and a day-case surgery in high-volume centers, without compromising safety and efficacy [10, 11]. In fact, mini-PCNL (mPCNL) has become the preferred surgical technique among endourologists for the treatment of kidney stones [11]. In this setting, urinary obstruction, due to postoperative edema, is a serious complication after mPCNL is performed without internal drainage, leading to increased hospitalization and eventually upper urinary tract stenting. The identification of clinical factors associated with postoperative lack of ureteral canalization would be of primary clinical importance to select the best candidate for indwelling stenting after mPCNL procedures.

Therefore, we conducted this cross-sectional, real-life study, with the aim to investigate the prevalence of and potential predictors of transient ureteral obstruction in a cohort of patients treated with mPCNL for kidney stones.

## Materials and methods

### Study cohort

We conducted a retrospective analysis of all consecutive patients who underwent mPCNL for renal stones in our tertiary referral academic center between January 2016 and November 2022. Collected data included patients’ anthropometrics and medical history. Comorbidities were scored with the Charlson comorbidity index (CCI) [12]. The diagnosis of urolithiasis was based on a preoperative urographic computerized tomography (CT) scan, which was used for the estimation of stone density (HU) and stone location. The stone volume was calculated using the ellipsoid formula (length × width × height × π × 1/6) [13]. A preoperative bladder urine culture was required in each case. One-shot parenteral prophylaxis was administered in case of negative culture. Patients with asymptomatic bacteriuria started a targeted therapy 48 and 72 h before the intervention. In cases of leukocytosis, urinary symptoms, or fever, the surgery was postponed after a full antibiotic course and negative urine culture.

### Surgical techniques

All procedures were performed by two expert endourologists (E.M; F.L) under general anesthesia with the patient in the supine Valdivia position. The surgical equipment included the MIP 16 F metallic sheath and dilator and the 16 F ClearPetra set (namely vamPCNL), the 12F MIP nephroscope, and the holmium laser (VersaPulse PowerSuite 100W; Lumenis, Israel) [14]. The procedure started with ureteral catheterization followed by a retrograde pyelography to assess the pelvicalyceal anatomy. Renal puncture was performed under combined fluoroscopic/ultrasonographic control. Tract dilatation was performed in one shot [15] with the MIP 16F metallic dilator or with the ClearPetra sheath assembled with its stylet. Irrigation was performed with a saline gravity bag suspended 1.5 m above the patient. Stone fragmentation was executed employing a 550-µm holmium:YAG laser fiber, configured in a short-pulse mode, with an energy range of 1.2 to 1.5 J and a frequency between 20 and 30 Hz, tailored to achieve stone fragmentation in accordance with surgical requirements. Stone fragments were removed using the vacuum cleaner effect during MIP (namely vcmPCNL) or through the aspiration-assisted sheath during vamPCNL. A flexible ureteroscope (7.9F; Olympus URF-P6, Germany) and nitinol baskets were used through the percutaneous access when residual fragments could not be removed with the mentioned devices. An 8F nephrostomy tube was used as the only exit strategy in all cases, while the ureteral catheter was removed immediately after surgery.

### Intraoperative and postoperative data

The number of percutaneous tracts and operative time (OT—time from placement of the ureteral catheter until its removal) were recorded. According to our internal protocol, uncomplicated procedures were managed as follows: The bladder catheter was removed on postoperative day 1, and the nephrostomy tube was closed; on postoperative day 2 (POD2), a percutaneous pyelography was performed to assess ureteral canalization. With the patient in a standing position, a vial of contrast medium was connected to the nephrostomy tube and injection was performed with gravity pressure. If immediate ureteral canalization was not observed, the tube was closed, the patient was invited to walk, and a plain X-ray was performed to check urine flow thereafter. When ureteral canalization was confirmed, the nephrostomy tube was removed. Patients were discharged on postoperative day 3. Patients with failed antegrade ureteral canalization were managed with observation or medications (steroids) for 24–48 h, and a second pyelography was performed before nephrostomy tube removal.

Postoperative complications were graded according to the PCNL-adjusted Clavien score [16, 17]. Patients were evaluated within 3 months after surgery with non-contrast-enhanced CT scan to identify residual stones [18]. Procedures were defined as stone-free if no residual stones were detected.

We excluded patients with renal or skeletal anomalies (*N* = 22); scheduled staged procedures for large stone burden (*N* = 44); significant intraoperative bleeding requiring indwelling stent or prolonged nephrostomy tube stay (*N* = 5); and endoscopic combined intrarenal surgery procedures (*N* = 3). A final cohort of 263 patients who underwent mPCNL for kidney stones was considered for statistical analysis.

Data collection adheres to the principles of the Declaration of Helsinki. All patients signed an informed consent agreeing to share their own anonymous information for future studies. The study was approved by the Foundation IRCCS Ca’ Granda—Ospedale Maggiore Policlinico Ethical Committee (Prot. 25,508).

### Statistical analysis

The distribution of data was tested with the Shapiro–Wilk test. Data are presented as medians (interquartile range; IQR) or frequencies (proportions). Descriptive statistics were used to describe the whole cohort. First, the rate of lack of antegrade ureteral canalization on POD2 was recorded. Second, clinical parameters and intraoperative and postoperative characteristics were compared between participants with ureteral canalization and those with failed antegrade ureteral canalization with the Mann–Whitney test and Fisher exact test, as indicated. Lastly, univariable and multivariable logistic regression models tested the association between clinical variables and ureteral obstruction on POD2. Statistical analyses were performed using SPSS v.26 (IBM Corp., Armonk, NY, USA). All tests were two-sided, and statistical significance level was determined at *p* < 0.05.

## Results

Overall, the median (IQR) age and BMI were 56 (47–65) years and 24.3 (21.7–27.5) kg/m^2^, respectively. A CCI ≥ 1 was found in 97 (36.8%) participants. Median stone volume was 1.7 (0.8–4.2) cm^3^, and 49% of patients had multiple stones (Table 1). Stones were located in the renal pelvis in 149 (56.7%) cases. vamPCNL and vcmPCNL were performed on 210 (79.8%) and 53 (20.2%) participants, respectively. Median operative time and hospitalization time were 100 (75–144) minutes and 4 (3–6) days. Lack of antegrade flow on POD2 was detected in 55 (20.9%) patients. After surgery, 225 (85.5%) patients were stone-free and 62 (23.5%) had postoperative complications (any Clavien) (Table 1).

**Table 1:** Demographic characteristics of the whole cohort (*n* = 263)

Age (years)	
Median (IQR)	56 (47–65)
Range	19–84
Male gender [no. (%)]	144 (54.8)
BMI (kg/m^2^)	
Median (IQR)	24.3 (21.7–27.5)
Range	17.9 – 42.2
CCI (score)	
Median (IQR)	0.0 (0.0)
Mean (SD)	0.7 (0.2)
Range	0–8
CCI ≥ 1 [no. (%)]	97 (36.8)
Laterality [no. (%)]	
Right	127 (48.2)
Left	136 (51.8)
Stone volume (cm^3^)	
Median (IQR)	1.7 (0.8–4.2)
Range	0.5 – 23.6
Multiple stones [no. (%)]	129 (49.0)
Stone in renal pelvis [no. (%)]	149 (56.7)
Mean stone density (Hounsfield unit)	
Median (IQR)	849 (590–1050)
Range	150 – 1983
Multiple access tracts [no. (%)]	38 (26.2)
Procedure type [no. (%)]	
vamPCNL	210 (79.8)
vcmPCNL	53 (20.2)
Multiple access tracts [no. (%)]	29 (11.0)
Operative time (min)	
Median (IQR)	100 (75–144)
Range	30 – 180
Lack of ureteral canalization [no. (%)]	55 (20.9)
Hospitalization time (days)	
Median (IQR)	4 (3–6)
Range	3 – 20
Postoperative complications [no. (%)]	
(Highest Clavien score)	
Clavien–Dindo I-II	50 (19.0)
Clavien–Dindo IIIa/b	12 (4.6)
Stone-free rate [n. (%)]	225 (85.5)

Keys: *BMI* body mass index, *CCI* Charlson Comorbidity Index, *vamPCNL* vacuum-assisted mini-percutaneous nephrolithotomy, *vcmPCNL* vacuum cleaner mini-percutaneous nephrolithotomy

Patients with failed antegrade canalization had larger stone volume [3.1 (1.1–5.6) cm^3^ vs. 1.8 (0.8–3.8) cm^3^, *p* < 0.01] and higher rate of multiple stones (61.8% vs. 45.7%, *p* = 0.03) than those without obstruction (Table 2). Operative time was longer [105 (75–140) min vs. 90 (73–125) min, *p* < 0.01], and stones were more frequently located in the renal pelvis (74.5% vs. 51.9%, *p* < 0.01) in cases with failed canalization. Conversely, the type of surgery (vamPCNL or vcmPCNL) did not affect canalization status. A longer length of stay (6 vs. 4 days, *p* < 0.01) and higher rate of postoperative complications (*p* = 0.03) were more frequently found in patients without ureteral canalization on POD2 (Table 2). Observation and steroids were used to treat postoperative edema in 69.1% and 30.9% of cases, respectively. The nephrostomy tube was removed earlier in patients treated with steroids than observation (3 vs. 4 days, *p* = 0.01).

**Table 2:** Descriptive statistics of the cohort as segregated according to ureteral canalization (*n* = 263)

	Ureteral canalization	Lack of canalization	*p*-value*
Age (years)			0.08
Median (IQR)	56 (47–64)	53 (47–65)	
Range	19–84	23–81	
Male gender [no. (%)]	113 (54.3)	31 (56.3)	0.8
BMI (kg/m^2^)			0.6
Median (IQR)	24.2 (21.5–28.1)	25.3 (22.6–26.7)	
Range	17.9–42.2	18.7–38.3	
CCI (score)			0.9
Median (IQR)	0.0 (0.0)	0.0 (0.0)	
Mean (SD)	0.6 (0.2)	0.6 (0.2)	
Range	0–8	0–6	
CCI ≥ 1 [no. (%)]	76 (36.5)	21 (38.1)	0.4
Laterality [no. (%)]			0.8
Right	100 (48.1)	27 (49.1)	
Left	108 (51.9)	28 (50.9)	
Stone volume (cm^3^)			< 0.01
Median (IQR)	1.8 (0.8–3.8)	3.1 (1.1–5.6)	
Range	0.5–18.9	2.7–23.6	
Multiple stone [no. (%)]	95 (45.7)	34 (61.8)	0.03
Stone in renal pelvis [no. (%)]	108 (51.9)	41 (74.5)	< 0.01
Mean stone density (Hounsfield unit)			0.5
Median (IQR)	825 (574–1080)	850 (629–1104)	
Range	150–1280	372–1983	
Multiple access tracts [no. (%)]	22 (10.6)	7 (12.7)	0.7
Procedure type [no. (%)]			0.4
vamPCNL	164 (78.8)	46 (83.6)	
vcmPCNL	44 (21.2)	9 (16.4)	
Operative time (min)			< 0.01
Median (IQR)	90 (73–125)	105 (75–140)	
Range	30–180	50–180	
Hospitalization time (days)			< 0.01
Median (IQR)	4 (3–5)	6 (4–7)	
Range	3–20	3–15	
Postoperative complications [no. (%)]			0.03
(Highest Clavien score)			
Clavien–Dindo I-II	35 (16.8)	15 (27.2)	
Clavien–Dindo IIIa/b	15 (7.2)	7 (12.7)	
Stone-free rate [no. (%)]	178 (85.5)	47 (85.4)	0.2

Keys: *BMI* body mass index, *CCI* Charlson Comorbidity Index, *vamPCNL* vacuum-assisted mini-percutaneous nephrolithotomy, *vcmPCNL* vacuum cleaner mini-percutaneous nephrolithotomy

^*^ *p*-value according to the Mann–Whitney test and Fisher exact test, as indicated

Logistic regression models were employed to assess potential predictors of ureteral obstruction on postoperative POD2. At univariable analysis, stone volume (OR 1.2, *p* = 0.02), renal pelvis location (OR 3.2, *p* = 0.001), and operative time (OR 1.2, *p* = 0.02) were associated with negative canalization (Table 3). At multivariable logistic regression analysis, higher stone volume (OR 1.1, *p* = 0.02) and renal pelvis location stones (OR 2.2, *p* = 0.04) were found to be independent predictors of ureteral obstruction on POD2, after accounting for operative time (Table 3).

**Table 3:** Logistic regression models predicting lack of ureteral canalization after mPCNL

	OR	*p*-value	95% CI		OR	*p*-value	95% CI
UVA model				MVA model			
Age	0.9	0.8	0.96–1.06				
BMI	1.1	0.2	0.97–1.11				
CCI ≥ 1	1.1	0.5	0.83–1.52				
Stone volume	1.2	0.02	1.02–1.38		1.1	0.02	1.01–1.42
Stone density	0.8	0.6	0.80–1.01				
Stone in renal pelvis	3.2	0.001	1.26–4.12		2.2	0.04	1.41–4.29
vcmPCNL vs	0.9	0.3	0.87–1.19				
vamPCNL							
Operative time	1.2	0.02	1.01–1.38		1.1	0.09	0.99–1.21

Keys: *UVA* univariate model, *MVA* multivariate model, *BMI* body mass index, *CCI* Charlson Comorbidity Index, *vamPCNL* vacuum-assisted mini-percutaneous nephrolithotomy, *vcmPCNL* vacuum cleaner mini-percutaneous nephrolithotomy

## Discussion

This study was specifically designed to investigate potential clinical factors associated with transient ureteral obstruction after mPCNL for kidney stones. We found that one out of five patients had impaired ureteral canalization on POD2 after mPCNL. Stone volume and stone location in the renal pelvis were found to be independent predictors for postoperative ureteral obstruction. As expected, patients with this complication had longer hospitalization time and higher rate of adverse events compared to those with the regular postoperative course. Of note, steroid treatment seemed to be associated with faster ureteral canalization as compared to observation.

Our study was motivated by the lack of standardized indications in terms of exit strategies and their postoperative management in patients treated with mPCNL. Due to the miniaturization and the technological improvement of the instruments, mPCNL has become a widely used procedure associated with low complications and good stone-free rate [19]. Consequently, tubeless and totally tubeless procedures, along with day-case procedures, are more frequently performed [10]. Nonetheless, early postoperative complications can occur even after uneventful mPCNL, thus neutralizing the advantages of a tubeless procedure or an early discharge [17]. Among these, transient upper urinary tract obstruction due to post-PCNL edema, in patients without urinary drainage, can cause severe pain, kidney function impairment, and hospital readmission [7]. For this reason, it is of primary clinical importance to identify patients at higher risk of ureteral obstruction, in order to improve the selection of totally tubeless mPCNL procedures.

In our study, 20% of patients treated with mPCNL showed ureteral obstruction during percutaneous pyelography on POD2. This rate is lower compared to that reported in a series of 241 standard PCNL, in which Authors found that 31.5% of patients had impaired ureteral canalization on POD1 [7]. Our results might differ from those in published literature for several reasons. First, Lee et al. [7] investigated antegrade flow by fluoroscopic antegrade nephrostogram, contrast-enhanced ultrasound, or methylene blue dye test. By using different tests, it is possible that the rate of ureteral obstruction might be different. Second, in our study, antegrade pyelography was performed on POD2, when it was expected that local edema might be lower compared to POD1. Third, mPCNL performed with miniaturized instruments might produce a lower damage to the pelvic parenchyma compared to standard procedures, thus limiting tissue manipulation and postoperative edema.

Persistent renal collecting system obstruction after PCNL is rare, but may result from stricture, infundibular stenosis, or avulsion which can further lead to hydronephrosis or hydrocalix [5]. Risk factors for prolonged obstruction after PCNL are longer operative time, large stone burden, and extended postoperative nephrostomy tube drainage [20]. Strictures after PCNL usually occur at the proximal ureter of the UPJ. Impaction of stone and also local trauma by intracorporeal lithotripsy are responsible for the stricture and obstruction [5]. Moreover, an increased risk for stricture formation is found in patients having a urinary diversion and proximal ureteral calculi due to an intense inflammatory response (obliterative pyeloureteritis) that may occur [20]. Therefore, routine postoperative imaging is mandatory to exclude silent obstructions after PCNL.

Transient ureteral obstruction after PCNL is more common, and it is primarily caused by intraoperative tissue manipulation causing local edema [7]. Several factors might be associated with intraoperative tissue damage and ureteral obstruction. Lee et al. [7] showed that mid or upper kidney access, ipsilateral ureteroscopy, and stones located anywhere other than in the renal pelvis were associated with an increased risk of lack of ureteral canalization on POD1 after standard PCNL. Kidney access to the mid and upper calyces makes the antegrade access to the UPJ and ureter easier, thus increasing the likelihood of mechanical trauma to the area during lithotripsy [7]. Similarly, concomitant ipsilateral ureteroscopy contributes to tissue instrumentation and subsequent local edema [7].

In this study, we found that larger stone volume and stone located in the renal pelvis were associated with a higher risk of ureteral obstruction after mPCNL. From a clinical standpoint, it could be speculated that with greater stone burden and operative time tissue instrumentation and consequent trauma would also increase, thus impairing postoperative ureteral canalization. Furthermore, the presence of stones in the renal pelvis is likely to cause more trauma at the UPJ level by itself and also during lithotripsy, generating local edema. Of note, clinical factors such as patient’s age, BMI, and stone density were not found to be associated with ureteral canalization. Similarly, ureteral obstruction was not associated with the device used during mPCNL (vcmPCNL or vamPCNL).

According to our Institutional protocol, patients with ureteral obstruction on POD2 were managed with observation or steroid treatment for 24–48 h according to physician preference, and a second pyelography was performed before nephrostomy tube removal. We showed that ureteral canalization was achieved earlier (3 vs. 4 days) in patients treated with steroids rather than observation. Therefore, it is likely that steroid treatment can be useful to counteract tissue edema and inflammation after mPCNL, promoting prompt ureteral canalization.

This study is innovative since it is the first with the aim of investigating rates of and predictors of ureteral obstruction in a relatively large cohort of patients treated with mPCNL for kidney stones. The first strength of this investigation is that each patient underwent a comprehensive diagnostic work-up and a standardized postoperative management with antegrade pyelography to check ureteral canalization. Conversely, previous studies used a combination of different procedures (contrast-enhanced ultrasound, methylene blue dye test, antegrade pyelography) to investigate urine flow, thus including potential variability in the rate of ureteral obstruction [7]. Second, differently from previous reports [7], our cohort of mPCNL did not include additional procedures such as ureteroscopy. Ureteroscopy is a potential contributor to tissue instrumentation and damage, leading to postoperative edema, but it is not a routine step of PCNL. Therefore, our study has a strong characterization in the everyday clinical practice by only including standard mPCNL procedures. Third, our results have important clinical implications in terms of exit strategy indications for mPCNL. It could be speculated that after mPCNL with large stones located in the renal pelvis, an indwelling ureteral catheter can be used to avoid postoperative ureteral obstruction and related complications.

Our study is not devoid of limitations. The retrospective nature of our investigation does not allow to draw general conclusions in terms of predictors and preventive strategies for ureteral obstruction after mPCNL. Also, this was a single-center-based study, which raises the possibility of selection biases; thereof, larger studies across different centers and cohorts are needed to externally validate our findings. Furthermore, our results are based on an institutional protocol for the postoperative management of patients treated with mPCNL, which can differ among stone centers. Therefore, our results cannot be generalized. Lastly, we only included patients treated with Holmium laser, but we cannot exclude that different energy modalities (pneumatic, ultrasonic) might impact ureteral patency.

## Conclusions

One out of five patients had transient ureteral obstruction after mPCNL for kidney stones in the real-life setting. Larger stone volume and stone location in the renal pelvis were found to be independent predictors for postoperative ureteral obstruction. Steroid treatment seemed to be associated with faster ureteral canalization as compared to observation. The identification of risk factor for postoperative ureteral obstruction is of primary clinical importance in order to select the best candidate for kidney drainage or totally tubeless mPCNL.

## Data Availability

Data is available upon request to the corresponding author.
